# Supplementary material for: Chiral Porphyrin Assemblies Investigated by a Modified Reflectance Anisotropy Spectroscopy Spectrometer
Source: Molecules. 2023 Apr 14;28(8):3471. doi: 10.3390/molecules28083471 (PMC10142836; doi:10.3390/molecules28083471)
Supplement: Supplementary file 1 [file molecules-28-03471-s001.zip › molecules-2301478-Supplementary Material.pdf]

Supplementary Material for

Chiral porphyrin assemblies investigated by a modified reflectance anisotropy spectroscopy spectrometer.

I. Tomei, B. Bonanni, A. Sgarlata, M. Fanfoni, R. Martini, I. Di Filippo, G. Magna, M. Stefanelli, D. Monti, R. Paolesse and C. Goletti

• **A) Optical spectra of samples whose CD-RAS spectra have been reported in the main text.**

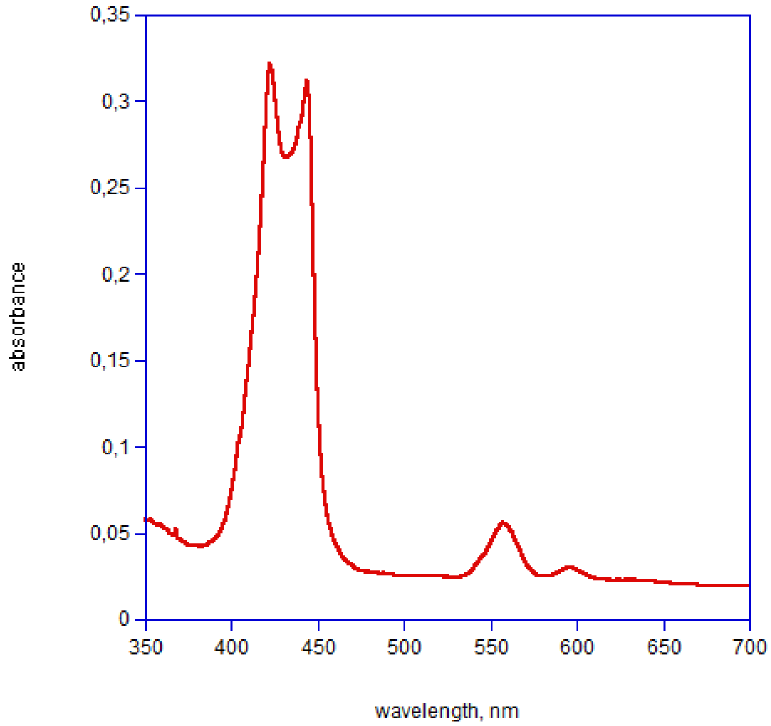

Figure S1: UV-Vis spectra of sample A1. The Soret band presents a double-peaked coupled B band (λmax = 422 and 443 nm) [Front. Chem., 15 October 2020 Sec. Supramolecular Chemistry Volume 8 - 2020].

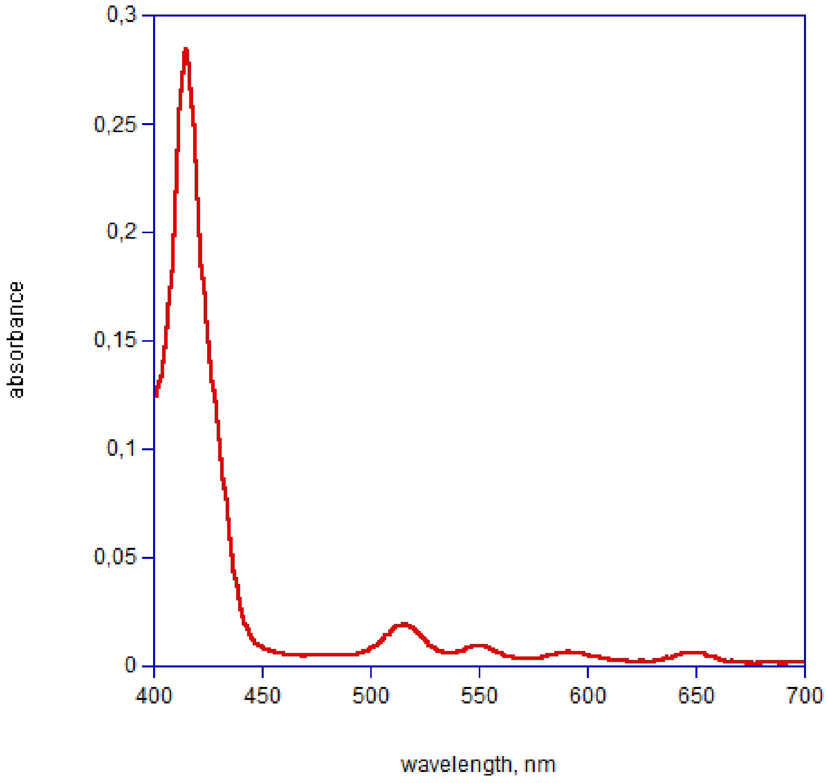

• **Figure S2:** UV-Vis spectra of sample A2.

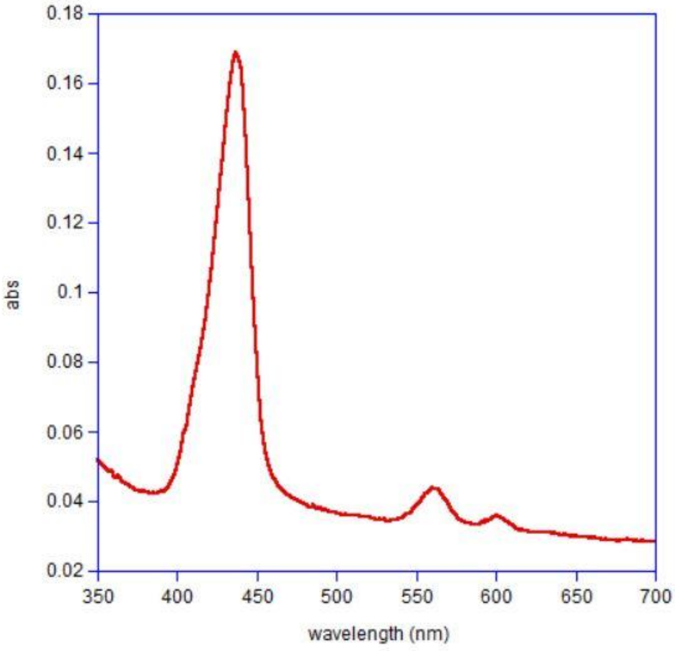

• **Figure S3:** UV-VIS spectra of sample C1.

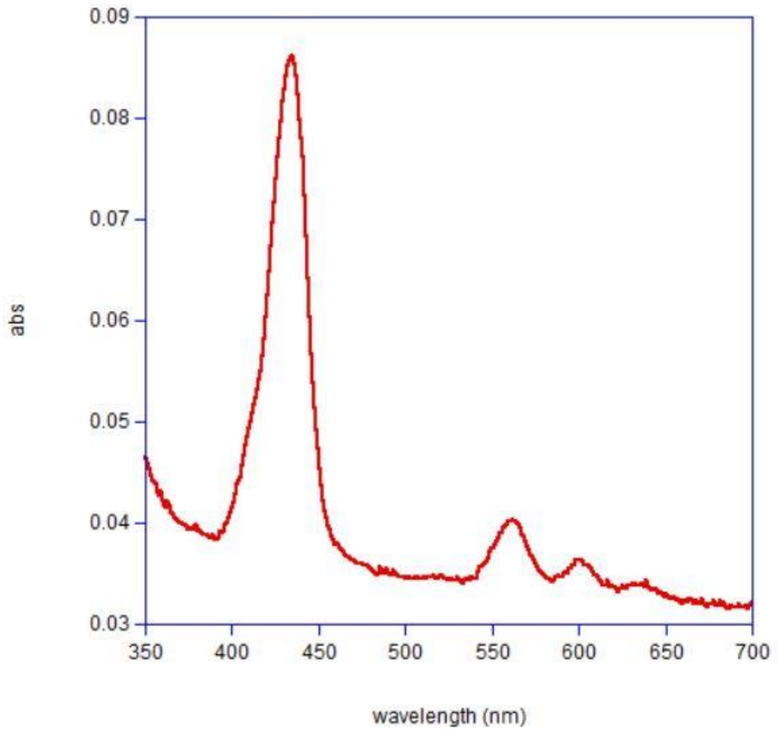

• **Figure S4:** UV-VIS spectra of sample C2.

• **B) Rationalization of the relation between the commercial CD unit and the RAS-CD.**

Here we describe how the data obtained by CD-RAS compare with the ones obtained by a commercial spectrometer. In fact, while CD-RAS data represent the intensity modulation of the signal when passing from the right circularly polarized state to the left circularly polarized state (see formula 2 in the main text), a commercial spectrometer measures the molar ellipticity due to the sample, that is the composition (generally associated to the elliptic polarization of light) of the two circularly polarized states, with the same intensity when impinge on the sample, but then differently absorbed. The molar ellipticity $[\theta]$ is defined as:

$$[\theta] = 4500\,\pi\,(\varepsilon_L - \varepsilon_R)\log_e 10 \qquad (S.1)$$

where ε_L and ε_R are the molecular extinction coefficients for light left and right handed polarized, respectively.

We define $\Delta\varepsilon$ as the difference between the molecular extinction coefficients for light left and right handed polarized light:

$$\Delta\varepsilon \equiv \varepsilon_L - \varepsilon_R \qquad (S.2)$$

$\Delta\varepsilon$ is estimated as:

$$\Delta\varepsilon \equiv \varepsilon_L - \varepsilon_R = \frac{1}{LC}\log_{10}\left(\frac{I_R}{I_L}\right) \qquad (S.3)$$

where L is the thickness of the absorbing layer (in cm), C is the molar concentration of the solute (in mol/liter), I_R and I_L are respectively the intensity of the beams for circularly polarized light left and right after it has been transmitted through the sample. By using (S.3) into (S.1), we obtain:

$$[\theta] = \frac{4500}{\pi}(\varepsilon_L - \varepsilon_R)\log_e 10 = \frac{4500}{\pi}(\Delta\varepsilon)\log_e 10 = \frac{4500}{\pi LC}\log_e 10 \log_{10}\left(\frac{I_R}{I_L}\right) \quad \text{(S.4)}$$

If $I_R/I_L \sim 1$ (a constraint satisfied for low absorption values), and defining:

$$I_A = \frac{I_R + I_L}{2}$$

$$S = I_R - I_L$$

after simple passages we obtain for the molar ellipticity $[\theta]$:

$$[\theta] = \frac{4500}{\pi LC}\left(\frac{S}{I_A}\right) = \frac{4500}{\pi LC}\,\frac{I_R - I_L}{\frac{I_R + I_L}{2}}$$

As $\Delta I/I = 2 \times (I_R - I_L)/(I_R + I_L)$, we conclude that the molar ellipticity $[\theta]$ and the $\Delta I/I$ resulting from a CD-RAS experiment are proportional:

$$[\theta] \sim \Delta I/I$$

(This part is a partially re-elaborated version of the Section 1 "Principles of operation" from the Manual for the JASCO CD Spectrometer Model J-1100/1500, see page 1).
